# Impact of Not Addressing Partially Cross-Classified Multilevel Structure in Testing Measurement Invariance: A Monte Carlo Study

**DOI:** 10.3389/fpsyg.2016.00328

**Published:** 2016-03-23

**Authors:** Myung H. Im, Eun S. Kim, Oi-Man Kwok, Myeongsun Yoon, Victor L. Willson

**Affiliations:** ^1^American Institutes of ResearchWashington, DC, USA; ^2^Department of Educational Psychology, Texas A&M UniversityCollege Station, TX, USA; ^3^Department of Educational and Psychological Studies, University of South FloridaTampa, FL, USA

**Keywords:** cross-classified multilevel data, measurement invariance, multilevel confirmatory factor analysis, cross-classified MIMIC, non-hierarchical structure data, simulations, Monte Carlo

## Abstract

In educational settings, researchers are likely to encounter multilevel data with cross-classified structure. However, due to the lack of familiarity and limitations of statistical software for cross-classified modeling, most researchers adopt less optimal approaches to analyze cross-classified multilevel data in testing measurement invariance. We conducted two Monte Carlo studies to investigate the performances of testing measurement invariance with cross-classified multilevel data when the noninvarinace is at the between-level: (a) the impact of ignoring crossed factor using conventional multilevel confirmatory factor analysis (MCFA) which assumes hierarchical multilevel data in testing measurement invariance and (b) the adequacy of the cross-classified multiple indicators multiple causes (MIMIC) models with cross-classified data. We considered two design factors, intraclass correlation (ICC) and magnitude of non-invariance. Generally, MCFA demonstrated very low statistical power to detect non-invariance. The low power was plausibly related to the *underestimated* factor loading differences and the *underestimated* ICC due to the redistribution of the variance component from the ignored crossed factor. The results demonstrated possible incorrect statistical inferences with conventional MCFA analyses that assume multilevel data as hierarchical structure for testing measurement invariance with cross-classified data (non-hierarchical structure). On the contrary, the cross-classified MIMIC model demonstrated acceptable performance with cross-classified data.

## Introduction

In educational and other social science research, multilevel data are commonly encountered. Although studies have investigated a variety of methodological issues related to multilevel modeling, such studies primarily have been limited to hierarchical linear models (Bell et al., 2013). Hierarchical linear models (HLMs) assume that in multilevel data, the levels are strictly nested or hierarchical, which means that a lower-level observation belongs to one and only one higher-level cluster. For example, in education settings, a student belongs to only a particular classroom while that classroom belongs to only a particular school. However, multilevel data may not always have a strictly nested or hierarchical structure, especially in education settings. For example, students are more likely to be nested within the schools they attend and the neighborhoods where they live at the same time, while schools and neighborhoods are not nested within each other. Instead, schools and neighborhoods are cross-classified with each other at the same level. This type of non-hierarchical multilevel data is also called *cross-classified* multilevel data. The use of cross-classified multilevel models has become more frequent in empirical research (e.g., Fielding, 2002; Jayasinghe et al., 2003; Marsh et al., 2008). On the other hand, some researchers did not fully consider the cross-classified structure of the data by simply ignoring one of the cross-classified factors in the data and used HLMs in their analyses (e.g., George and Thomas, 2000; Ma and Ma, 2004).

With increased understanding of the importance of proper analytic approach for cross-classified multilevel data (Goldstein, 1986, 1995; Rasbash and Goldstein, 1994; Raudenbush and Bryk, 2002), many major multilevel modeling textbooks have introduced techniques for handling cross-classified multilevel data such as cross-classified random effect modeling (CCREM) that can be specified in various multilevel modeling computer programs (e.g., HLM, SAS, MLwiN, and R). However, research examining the impact of misspecifying cross-classified multilevel data as strictly hierarchical multilevel data in different analytical settings such as structural equation modeling (SEM) has been quite limited.

A few methodological investigations have been conducted to examine the effects of misspecifing cross-classified multilevel data as strictly hierarchical multilevel data by ignoring one of the crossed factors in linear regression modeling (Berkhof and Kampen, 2004; Meyers and Beretvas, 2006; Luo and Kwok, 2009). In general, these studies have found that not fully taking a cross-classified multilevel data structure into account (i.e., treating the cross-classified data as strictly hierarchical by ignoring a crossed factor) can cause bias in variance component estimates, which results in biased estimation of the standard errors of parameter estimates. Ultimately, this may lead to incorrect statistical conclusions. In particular, Luo and Kwok's (2009) simulation study found that under the situation in which the crossed factors were completely cross-classified (i.e., nonzero ICCs associated with the factors), all variance components from the ignored crossed factor at the higher level were redistributed/added to the variance component at the lower level (i.e., overestimated variance component) while the variance component of the remaining crossed factor was underestimated.

Testing measurement invariance (MI) has become increasingly common in social science research when a measure is used across subgroups of a population or different time points of repeated measures. MI holds when persons of the same ability on a latent construct have the identical probability of obtaining a given observed score regardless of the group membership (Meredith and Millsap, 1992). Testing measurement invariance is a very important step before one can meaningfully compare the (mean) difference on a latent construct or the corresponding composite score between groups. The use of a measure with measurement bias (i.e., non-invariance) might lead to invalid comparisons. In other words, when measurement invariance is violated, observed differences in latent constructs or composite scores between subgroups or across time are ambiguous and difficult to interpret (Meredith and Teresi, 2006). Therefore, it is important to confirm that the scale we use measures the same latent construct (or has exactly the same meaning) across the groups we intend to compare.

Although many researchers have discussed the importance of establishing measurement invariance and the practical impact of measurement bias (Widaman and Reise, 1997; Borsboom, 2006; Meredith and Teresi, 2006; Yoon and Millsap, 2007; Fan and Sivo, 2009), there is very limited research on measurement invariance in multilevel data with non-hierarchical structure. For measurement invariance testing with hierarchical multilevel data, multilevel confirmatory factor analysis (MCFA; Mehta and Neale, 2005; Kim et al., 2012a) is widely used. However, in reality, multilevel data may not always have a strictly hierarchical structure, particularly in research situations where lower-level observations are nested within multiple higher-level clusters (e.g., schools and neighborhoods or items and raters) that are cross-classified simultaneously at the same level. When levels of multilevel data are cross-classified, conventional multilevel modeling cannot be used to model the cross-classified clustering effects. Because conventional multilevel modeling cannot account for the effects of multiple cluster factors simultaneously when these multiple cluster unit factors are crossed at the same level, it treats cross-classified multilevel data as strictly nested multilevel data by ignoring one of the crossed factors (e.g., either schools or neighborhoods; items or raters) in the analysis.

In addition, the current capability of commonly available SEM software does not permit multiple-group comparison along with multilevel confirmatory factor analysis (MCFA) for cross-classified multilevel data yet, which is a critical feature for testing measurement invariance. Although many researchers understand the importance of establishing measurement invariance for a measure and conducting a correct analysis for cross-classified multilevel structured data, they might treat the cross-classified multilevel data as hierarchical multilevel data by ignoring crossed factor(s) in measurement invariance testing under some circumstances such as the limitations/restrictions of current SEM software. Hence, it is important to examine the potential impact of ignoring the cross-classified data structure in conventional multilevel CFA. This study is the first to test measurement invariance with cross-classified multilevel data within multilevel SEM framework.

The primary purpose of the present study was to investigate the consequences of having non-invariance with cross-classified multilevel data. In the present study, we conducted two Monte Carlo studies. First, we examined the performance of MCFA (i.e., misspecified model) in detecting a non-invariant factor loading across between-level groups after treating a cross-classified data as strictly hierarchical multilevel data in Study 1. Second, we investigated the adequacy of the cross-classified multiple indicators multiple causes (MIMIC) model in detecting a non-invariant intercept across between-level groups by fitting a correct model in testing measurement invariance with cross-classified data in Study 2. Both studies focused on non-invariance at the between-level grouping. We examined the statistical power of measurement invariance testing and simulation factors that impacted the statistical power. Below we first briefly review the conventional MCFA and cross-classified MCFA for measurement invariance testing, followed by the research design and simulation study conditions.

## Materials and methods

### Comparison between conventional MCFA and cross-classified MCFA

**Figure 1:**
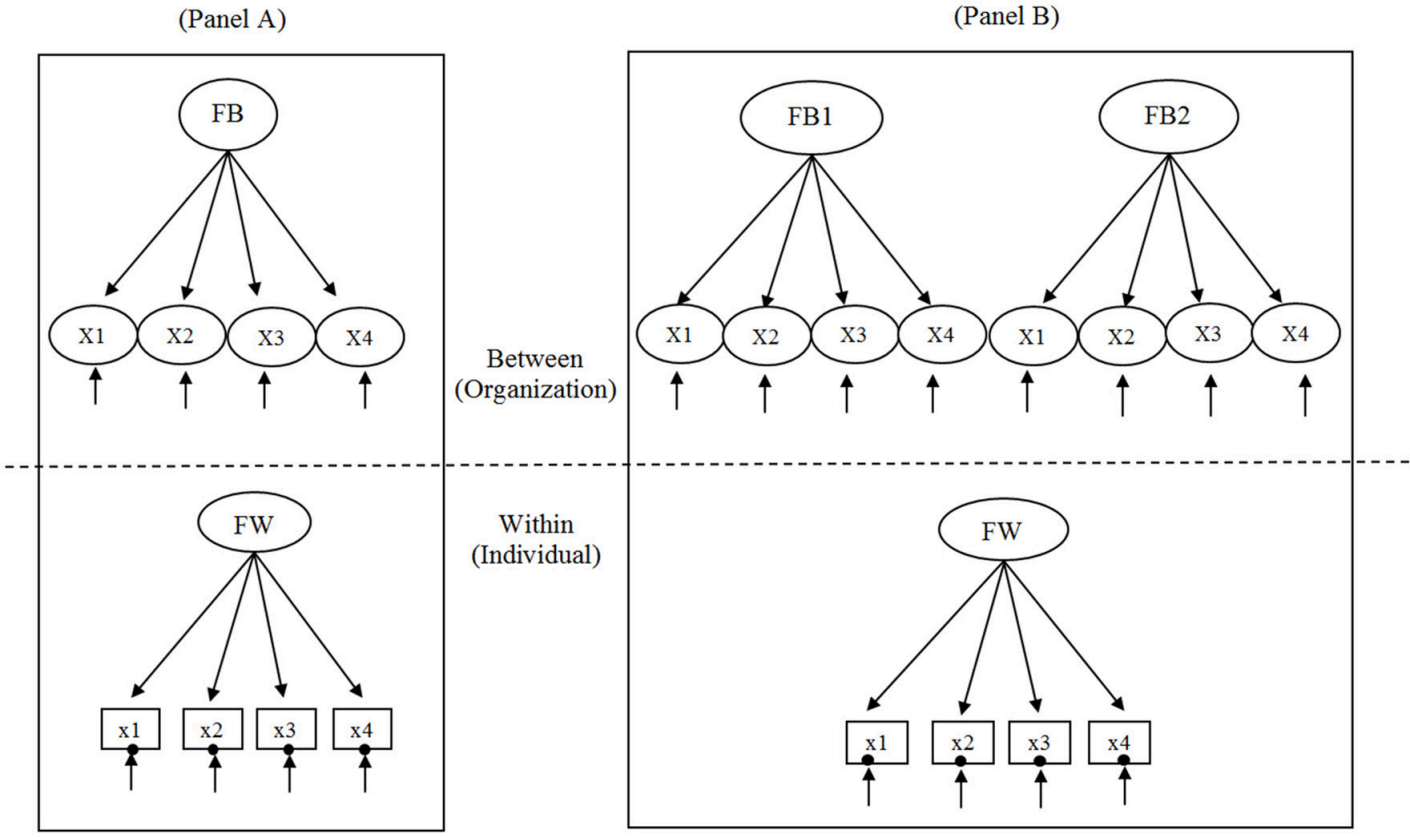
**Two-level conventional multilevel confirmatory factor analysis (MCFA) depicted in (A) and two-level cross-classified MCFA depicted in (B)**. FW is within-level latent factor; FB is between-level latent factor; FB1 and FB2 are the two crossed factors 1 and 2, respectively, at the between-level. In the within part of the model, XI–X4 are the continuous observed variables, and the random intercept is shown as a filled circle at the end of the arrow pointing to each observed variable.

The simplest two-level conventional MCFA and two-level cross-classified MCFA of a single factor with four observed variables used for the simulation study are illustrated in Figures 1A,B, respectively. For this study, we examine the two MCFAs for continuous variables. A summary table of the differences and similarities between conventional MCFA and cross-classified MCFA is provided in Supplementary Material. Consider the example of a two- level conventional MCFA in which students (*i*) are strictly nested within schools (*j*) and a two-level cross-classified MCFA in which students (*i*) are cross-classified by schools (j_1_) and neighborhoods (*j*_2_). Adopting the notation of Rasbash and Goldstein (1994), *X*_*i*(*j*_1_*j*_2_)_ refers to observed variables of student outcome where *i* indexes a within-level unit, *j*_1_ indexes a cluster of crossed factor of school (FB1), and *j*_2_ indexes a cluster of crossed factor of neighborhood (FB2).

The relation between the observed variables and the latent factors for MCFA and cross-classified MCFA can be expressed as Equations (1a) and (1b), respectively.

(1a) $$\begin{array}{r} X_{ij}=\tau +\;{\Lambda \eta }_{ij}+\;\varepsilon_{ij} \end{array}$$

(1b) $$\begin{array}{r} \;X_{i\left(j_{1}j_{2}\right)}=\tau +\;{\Lambda \eta }_{i\left(j_{1}j_{2}\right)}+\;\varepsilon_{i\left(j_{1}j_{2}\right)} \end{array}$$

where *X* is a matrix of observed scores, τ is a vector of intercepts, Λ is a matrix of factor loadings, η is a matrix of latent or common factor scores, and ε is a matrix of unique factor scores or residuals (Kaplan, 2009). It is assumed that the observed variables are multivariate normally distributed. In multilevel CFA, the assumption that observations are independent and identically distributed (Muthén, 1994) should be relaxed with the multilevel data where lower observations nested within higher-level clusters are dependent/correlated with each other.

By allowing random effects to vary across clusters, the latent factor scores (η_*ij*_) for MCFA can be decomposed into two parts (Equation 2a) whereas the latent factor scores (η_*i*(*j*_1_*j*_2_)_) for cross-classified MCFA can be decomposed into three parts (Equation 3b) as follows:

(2a) $$\begin{array}{r} \eta_{ij}=\alpha +\;\eta_{wij}+\;\eta_{Bj} \end{array}$$

(2b) $$\begin{array}{r} \eta_{i\left(j_{1}j_{2}\right)}=\alpha_{j_{1}}+\alpha_{j_{2}}\;+\;\eta_{wij}+\;\eta_{{Bj}_{1}}+\;\eta_{{Bj}_{2}} \end{array}$$

where α is the expected value or grand mean of η_*ij*_ and α_*j*_1__ and α_*j*_2__ are the expected values or mean of each crossed-factor of FB1 and FB2 at the between-level, respectively. η_*wij*_ is the individual effects for both models, whereas η_*B*_*j*__ is for one cluster effect for MCFA and η_*B*_*j*_1___ and η_*B*_*j*_2___ represent cluster effects of the crossed-factors FB1 and FB2, respectively for cross-classified MCFA.

In the same way, observed variable of *X*_*ij*_ and *X*_*i*(*j*_1_*j*_2_)_ can also be re-expressed into two parts for MCFA (Equation 4a), that is, within-level and between- level components and three parts for cross-classified MCFA(Equation 3b), that is, one within-level and two between-level components as,

(3a) $$\begin{array}{r} X_{ij}=\;\tau_{B}+{\;\Lambda_{W}\eta }_{Wij}\;+{\Lambda_{B}\eta }_{Bj}+\;\varepsilon_{wij}+\varepsilon_{Bj} \end{array}$$

(3b) $$\begin{array}{r} X_{i\left(j_{1}j_{2}\right)}=\tau_{{Bj}_{1}}+\tau_{{Bj}_{2}}{+\;\Lambda_{W}\eta }_{Wij}\;{{+\;\Lambda }_{{Bj}_{1}}\eta }_{{Bj}_{1}}+{\Lambda_{{Bj}_{2}}\eta }_{{Bj}_{2}}+\varepsilon_{{Bj}_{1}}\\ \;+\varepsilon_{{Bj}_{2}}+\varepsilon_{wij} \end{array}$$

where τ is the intercept of observed variable (*X*_*ij*_) at the cluster level.

For multilevel CFA, the intercept of an observed variable is only expressed with the intercept (τ_*B*_ for MCFA and τ_*B*_*j*_1___ and τ_*B*_*j*_2___ for cross-classified MCFA) at the between-level. This is because an individual score is the combination of the group mean and its deviation from the group means (Heck and Thomas, 2009). It should be noted that unlike the multilevel model for strictly hierarchical data, in which only one intercept (τ_*B*_) is estimated at the between-level for MCFA, two intercepts (τ_*B*_*j*_1___ and τ_*B*_*j*_2___ for FB1 and FB2, respectively) are estimated for cross-classified MCFA due to the two crossed factors of FB1 and FB2 at the between-level of an observed variable (*X*_*i*(*j*_1_*j*_2_)_).

Given that factor means vary across clusters as expressed in Equation (2), the variance of the factor can be partitioned into two components for conventional MCFA and three components for cross-classified MCFA as follows:

(4a) $$\begin{array}{r} V\;\left(\eta_{ij}\right)=\;\Psi_{T}=\;\Psi_{W}+\Psi_{B} \end{array}$$

(4b) $$\begin{array}{r} V\;\left(\eta_{i\left(j_{1}j_{2}\right)}\right)=\;\Psi_{T}=\Psi_{W}+\;\Psi_{{Bj}_{1}}+\;\Psi_{{Bj}_{2}} \end{array}$$

where Ψ_*B*_ is the between-level factor variance for MCFA and Ψ_*B*_*j*_1___ and Ψ_*B*_*j*_2___ are the between-level factor variances for the two crossed factors of FB1 and FB2 in cross-classified MCFA. Ψ_*W*_ is the within-level factor variance for both models, and Ψ_*T*_ is the total factor variance which is the sum of between-level and within-level factor variances. The ratio of the between variance to the total variance which is called intraclass correlation (ICC) can be viewed as an indicator of data dependency (Snijders and Bosker, 1994; Raudenbush and Bryk, 2002). For the total variability between two crossed factors, two variance components (i.e., Ψ_*B*_*j*_1___ and Ψ_*B*_*j*_2___ for FB1 and FB2, respectively) are summed up as suggested by Meyers and Beretvas (2006). Under the completely cross-classified situation (i.e., units in a cluster of one crossed factor can affiliate with any clusters of the other crossed factor and vice versa), the two crossed factors (i.e., complete cross-classification situation) are independent from each other. The ICC in MCFA (Equation 5a) and the two ICCs for the two crossed-factors in cross-classified MCFA can be estimated as follows:

(5a) $$\begin{array}{r} ICC\eta_{B}=\frac{\Psi_{B}}{\Psi_{W}+\Psi_{B}} \end{array}$$

(5b) $$\begin{array}{r} ICC\eta_{Bj1}=\frac{\Psi_{Bj1}}{\Psi_{W}+\Psi_{Bj1}+\Psi_{Bj2}},\\ ICC\eta_{Bj2}=\frac{\Psi_{Bj2}}{\Psi_{W}+\Psi_{Bj1}+\Psi_{Bj2}} \end{array}$$

where *ICC*_η*B*_ refers to the ICC for the between factor FB in MCFA while *ICC*_η*B*_*j*1__ and *ICC*_η*B*_*j*2__ refer to the ICCs for the two crossed factors FB1 and FB2 in cross-classified MCFA, respectively. It should be noted that we assume the equality of the factor loadings across the two factors at the between-level and the equality of factor loadings across the within- and between- levels (Rabe-Hesketh et al., 2004; Mehta and Neale, 2005). It is called factorial invariance across levels or cross-level factorial invariance. The variance of the unique factor or residual also comprises two elements for MCFA (Equation 6a); that is, between-level component and within-level component. Similarly, there ae three elements in the residual variance for cross-classified MCFA; that is, two between-level components and one within-level component (Equation 6b) as,

(6a) $$\begin{array}{r} V\;\left(\varepsilon_{ij}\right)=\;\Theta_{W}+\;\Theta_{B} \end{array}$$

(6b) $$\begin{array}{r} V\;\left(\varepsilon_{i\left(j_{1}j_{2}\right)}\right)=\;\Theta_{W}\;+\;\Theta_{{Bj}_{1}}\;+\Theta_{{Bj}_{2}} \end{array}$$

Finally, with the independence assumption between the common factor (η) and the unique factor (ε) as in a regular CFA (*Cov* (η, ε) = 0), the covariance structures of the MCFA (Equation 7a) and cross-classified MCFA (Equation 7b) are defined as follows:

(7a) $$\begin{array}{l} \Sigma_{B}=\Lambda_{B}\Psi_{B}\Lambda \prime_{B}+\Theta_{B,}\\ \Sigma_{W}=\Lambda_{W}\;\Psi_{W}\Lambda \prime_{W}+\Theta_{W,}\\ \Sigma_{T}=\Lambda_{W}\;\Theta_{W}\Lambda \prime_{W}+\Lambda_{B}\Psi_{B}\Lambda \prime B+\Theta_{W}+\Theta_{B} \end{array}$$

(7b) $$\begin{array}{l} \Sigma_{Bj_{1}}=\Lambda_{Bj_{1}}\Theta_{Bj_{1}}\Lambda \prime_{Bj_{1}}+\;\Theta_{Bj_{1}},\\ \Sigma_{Bj_{2}}=\Lambda_{Bj_{2}}\Psi_{Bj_{2}}\Lambda \prime_{Bj_{2}}+\;\Theta_{Bj_{2},}\\ \Sigma_{W}=\Lambda_{W}\Psi_{W}\Lambda \prime_{W}+\Theta_{W},\\ \Sigma_{T}=\Lambda_{W}\;\Psi_{W}\Lambda \prime_{W}+\Lambda_{Bj_{1}}\Psi_{Bj_{1}}\Lambda \prime_{Bj_{1}}+\Lambda_{Bj_{2}}\;\Psi_{Bj_{2}}\Lambda \prime_{Bj_{2}}\\ \;+\;\Theta_{W}+\Theta_{Bj_{1}}+\Theta_{Bj_{2}} \end{array}$$

Σ_*B*_ is the between-level variance matrix for MCFA whereas Σ_*B*_*j*_1___ and Σ_*B*_*j*_2___ are the between-level variance matrices of crossed-factor FB1 and FB2, respectively for cross-classified MCFA. Σ_*W*_ is the within-level variance matrix for both MCFAs, and Σ_*T*_ is the total variance matrix, which is the sum of within-level and between-level variance matrices.

### Factorial invariance in cross-classified multilevel data

One primary issue with assessing the measurement invariance in multilevel data (Curran, 2003; Mehta and Neale, 2005; Selig et al., 2008; Zyphur et al., 2008; Jones-Farmer, 2010; Kim et al., 2012a) is data dependency. This is of concern because with multilevel data the measurement bias (i.e., measurement non-invariance) across groups can be found at different levels, including the individual level, the cluster level (group or organizational level), or both. In other words, measurement invariance can be examined at different levels in multilevel data (Mehta and Neale, 2005), depending on the interest of measurement invariance. With the use of MCFA, we can test measurement invariance at different levels in multilevel data (Kim et al., 2012a).

Measurement invariance within a factor model is called factorial invariance. Factorial invariance (FI) can be represented within a linear factor model with mean and covariance structures whereas measurement invariance is a broad term that includes both linear and nonlinear relationships between observed variables and latent factors considering the entire score distribution (Yoon, 2008). Under the linear CFA framework, FI is defined as the equivalence of parameters specified in the model across groups. Thus, researchers check different levels of factorial invariance sequentially depending on the equivalent parameters in the testing of invariance. The levels of FI are discussed shortly.

Like FI testing with conventional multilevel data, the differences across groups can be found and tested at the individual-level, the organizational-level (cluster or between), or both with cross-classified multilevel data. Greater complexity might arise with FI testing of cross-classified multilevel data due to multiple higher level clusters (e.g., schools and neighborhoods; items and raters). Specifically, for the multiple higher level clusters, non-invariance can exist at each organizational-level cluster or both.

In general, FI testing can be conducted using a series of multiple-group CFA models, which impose identical parameters across groups. That is, the models that investigate the invariance of factor pattern (configural invariance), factor loadings (metric or weak invariance), latent intercepts (scalar or strong invariance), and unique factor or residual variances (strict invariance) are tested across groups in the sequential order. As discussed before, because cross-classified MCFA has multiple cluster-level crossed factors (e.g., students nested within schools and neighborhoods) compared to only one group-level factor in conventional CFA, for each between-level group comparison the separate FI testing should be conducted to detect the violation of invariance at the different between-level models across the different between-level comparison clusters.

To illustrate FI in cross-classified multilevel data, suppose that a grouping variable exists at the organizational level such as a treatment administered at schools, at neighborhoods, or at both crossed factors. With a grouping variable at the between-level, the two-level cross-classified MCFA (two crossed factors representing each cluster unit such as schools and neighborhoods at the same level as defined in Equation 1b through Equation 7b) can be directly expanded to multiple-group cross-classified MCFA by incorporating a group indicator as follows:

(8) $$\begin{array}{r} {\;X}_{i\left(j_{1}j_{2}\right)g}=\tau_{{Bj}_{1}g}+\tau_{{Bj}_{1}g}+{\Lambda_{{Bj}_{1}g}\eta }_{{Bj}_{1}g}+{\Lambda_{B_{2}g}\eta }_{{Bj}_{2}g}+{\Lambda_{Wg}\eta }_{Wijg}\\ \;+\varepsilon_{{Bj}_{1}g}+\varepsilon_{{Bj}_{2}g}+\varepsilon_{wijg},\\ \;{\;\Sigma }_{{Bj}_{1}g}={\;\Lambda_{{Bj}_{1}g}\Psi }_{{Bj}_{1}g}\Lambda_{{Bj}_{1}g}^{\prime }+\Theta_{{Bj}_{1}g},\\ \;\Sigma_{{Bj}_{2}g}={\;\Lambda_{{Bj}_{2}g}\Psi }_{{Bj}_{2}g}\Lambda_{{Bj}_{2}g}^{\prime }+\Theta_{{Bj}_{2}g,}\\ \;{\;\Sigma }_{Wg}=\;{\;\Lambda_{Wg}\;\Psi }_{Wg}{\;\Lambda }_{Wg}^{\prime }+\;\Theta_{wg} \end{array}$$

where a subscript, *g* is a group indicator (1, 2, …, G) and others are as described above.

First, configural invariance evaluates whether the groups of interest have equivalent patterns for the within-level model and the two between-level models (e.g., number of factors in within and between models and number of indicators for each factor). Second, when testing metric/weak invariance, the null hypotheses of the invariance of factor loadings, *H*_0Λ_, can be tested at both between-level crossed-factors FB1 and FB2 and at the within-level, respectively as such

(9) $$\begin{array}{r} H_{0\Lambda_{{Bj}_{1}}}:\;\Lambda_{{Bj}_{1}1}=\;\Lambda_{{Bj}_{1}2}=\cdots =\;\Lambda_{{Bj}_{1}G},\;\\ H_{0\Lambda_{{Bj}_{2}}}:\Lambda_{{Bj}_{2}1}=\;\Lambda_{{Bj}_{2}2}=\cdots =\;\Lambda_{{Bj}_{2}G},\\ H_{0\Lambda_{W}}:\;\Lambda_{W1}\;=\;\Lambda_{W2}\;=\cdots =\;\Lambda_{WG} \end{array}$$

Third, when testing scalar/strong invariance, the null hypotheses of the invariance of intercepts, *H*_0τ_, can be additionally tested only at the between-level crossed-factors FB1 and FB2, respectively as such

(10) $$\begin{array}{r} H_{0\tau_{{Bj}_{1}}}:\;\tau_{{Bj}_{1}1}=\;\tau_{{Bj}_{1}2}=\cdots =\;\tau_{{Bj}_{1}G},\\ {\;H}_{0\tau_{{Bj}_{2}}}:\;\tau_{{Bj}_{2}1}=\;\tau_{{Bj}_{2}2}=\cdots =\tau_{{Bj}_{2}G} \end{array}$$

Fourth, when testing strict invariance, the null hypotheses of the invariance of unique variances,*H*_0Θ_, can be tested at both between-level crossed-factors FB1 and FB2, and at the within-level, respectively as such

(11) $$\begin{array}{r} H_{0\Theta_{{Bj}_{1}}}:\;\Theta_{{Bj}_{1}1}=\;\Theta_{{Bj}_{1}2}=\cdots =\;\Theta_{{Bj}_{1}G},\\ {\;H}_{0\Theta_{{Bj}_{2}}}:\;\Theta_{{Bj}_{2}1}=\;\Theta_{{Bj}_{2}2}=\cdots =\Theta_{{Bj}_{2}G},\\ H_{0\Theta_{W}}:\;\Theta_{W1}\;=\;\Theta_{W2}\;=\cdots =\;\Theta_{WG} \end{array}$$

## Study 1: testing factorial invariance using multilevel confirmatory factor analysis (MCFA) with cross-classified data

### Methods

#### Data generation

Table 1 and Supplementary Material present the population values of parameters and M*plus* script, respectively, that were used for generating the cross-classified data.

**Table 1:** **Population parameters used for cross-classified multilevel data generation**.

**Level**	**ICC**	**DIF**	**Target factor loading**			**Factor variance**	**Unique variance**
			**G1**	**G2**	**Δ(G1-G2)**		
Between (FB1, school)	Small	Small		0.75	0.15	0.10	0.05
		Medium	0.90	0.65	0.25		
		Large		0.55	0.35		
	Medium	Small		0.75	0.15	0.25	0.05
		Medium	0.90	0.65	0.25		
		Large		0.55	0.35		
	Large	Small		0.75	0.15	0.50	0.05
		Medium	0.90	0.65	0.25		
		Large		0.55	0.35		
Between (FB2, neighborhood) ignored in the analysis	Small	Small				0.10	0.05
		Medium	0.90	0.90	None		
		Large					
	Medium	Small				0.25	0.05
		Medium	0.90	0.90	None		
		Large					
	Large	Small				0.50	0.05
		Medium	0.90	0.90	None		
		Large					
Within	Small	Small				1.00	0.25
		Medium	0.90	0.90	None		
		Large					
	Medium	Small				1.00	0.25
		Medium	0.90	0.90	None		
		Large					
	Large	Small				1.00	0.25
		Medium	0.90	0.90	None		
		Large					

*ICC is intra-class correlation. DIF is the difference in between-level factor loadings of the target item between groups. G1 is group 1 as a reference group; G2 is group 2 as a focal group where the factor loading of one item was set to be smaller than the factor loading of G1 in the study*.

##### Partial cross-classification

In order to mimic real educational settings, we created the partial cross-classification structure. As followed by Luo and Kwok's (2009) simulation study, we created 50 neighborhoods (FB2) nested within 20 schools (FB1) and students are cross-classified by schools and neighborhoods. In a full cross-classification, students from a specific school can live in any neighborhood and students from a specific neighborhood can go to any school. For example, all 50 neighborhoods are selected as cross-classified with all 20 schools. In reality, however, a partially cross-classified data condition in which students living in certain neighborhoods only go to certain schools and students attending certain schools only live in certain neighborhoods is more likely to occur.

**Figure 2:**
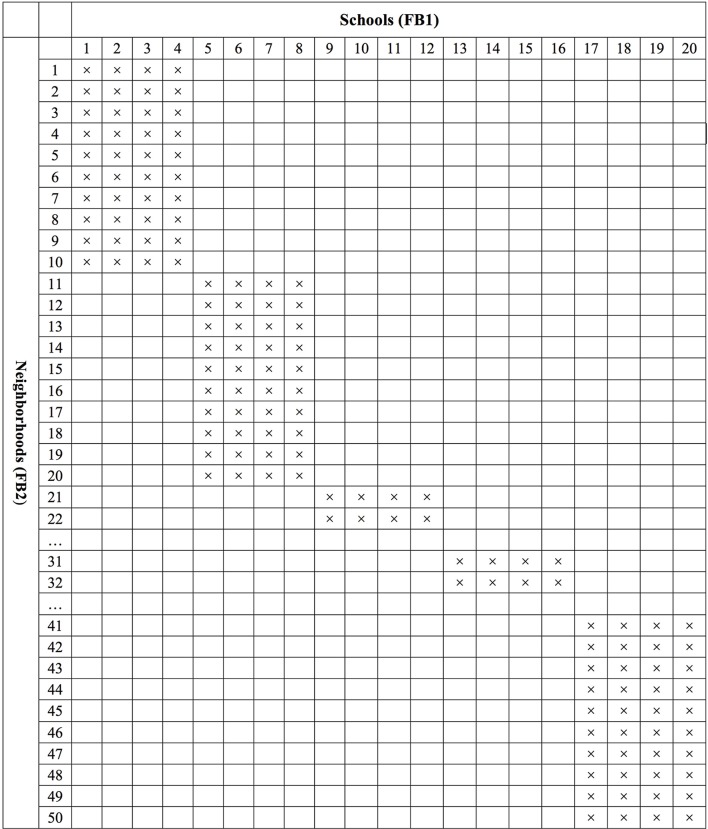
**Partial cross-classification data structure where students are cross-classified by schools (FB1) and neighborhoods (FB2)**.

In the current Monte Carlo study, a two-level fully cross-classified MCFA pertinent to Equation (2b) was first generated using M*plus* version 7.4 (Muthén and Muthén, 1998-2012). Then, we modified the generated full cross-classification structure data in order to create the partial cross-classification structure data (see Figure 2 for the structure). To create the partially cross-classified structure as shown in Figure 2, we first generated fully cross-classified data (50 neighborhoods × 20 schools × 20 students). Then, we selected the 10 neighborhoods in the sample and then designated only 4 schools for these 10 neighborhoods to create data in which students from these 10 neighborhoods go to the 4 designated schools not to any school (800 = 10 neighborhoods × 4 schools × 20 students). This process was repeated five times for all 50 neighborhoods, which yielded five blocks of subsets. Finally, these five blocks of subsets were combined. Under the partial cross-classified situation, a total of 4000 observations (i.e., 800 × 5 blocks) were generated for each simulated data set. We constructed a balanced design with two groups of equal size (i.e., 10 observations per group). This design reflects a common research situation in which two groups have similar sample sizes with an unknown direction of possible bias.

##### Factor structure

For the within and between models, we simulated a single factor with four factor indicators (DiStefano and Hess, 2005) of two groups while assuming factorial invariance across levels (i.e., the equivalence of factor loadings with the same number of factors across the between and within levels. For the population parameters, we referred to previous simulation studies on both measurement invariance and multilevel SEM (Hox and Maas, 2001; Maas and Hox, 2005; Yoon and Millsap, 2007; Kim et al., 2012b). In the present study, the factor loadings of the four items ranged from 0.7 through 1.0 at both levels (within-level and between-level, FB1 and FB2). The factor loading of one item is fixed at one for identification. The unique variances of the four observed variables were set to 0.25 for within-level (Hox and Maas, 2001) and 0.05 for between-level (see item-level ICC section below).

#### Simulation design factors

Two main design factors, namely, magnitude of the noninvariant factor loading and intraclass correlation (ICC), were considered. For the invariance testing, the target groups existed only at the between-level clusters (e.g., public and private schools). Thus, for the invariance (or 0 difference) condition, all parameters were set to be identical across groups. On the other hand, for the non-invariance condition, one of the between-level factor loadings was set to be different across groups only for the remaining crossed factor (e.g., schools).

The magnitude of difference in the target between-level factor loading (λ_*B*_*j*_1___) at the FB1 crossed factor between the two groups (i.e. the difference between λ_*B*_*j*_1___ for group 1 and the λ_*B*_*j*_2___ for group 2) was simulated at three levels: 0.15, 0.25, and 0.35 for small, medium and large differences, respectively where the group 1(G1) is a reference group and the group 2 (G2) is a focal group. The magnitude of difference was determined on the basis of the previous literature regarding the factorial invariance testing (Meade and Lautenschlager, 2004; Stark et al., 2006; French and Finch, 2008).

The intraclass correlation (ICC) was another design factor considered in this simulation study. Previous simulation studies have showed that the ICC could affect the statistical power for detecting non-invariance in MCFA, particularly at the between-level (Hox and Maas, 2001; Kim et al., 2012a). Based on previous simulation studies (Hox and Maas, 2001; Maas and Hox, 2005), three ICC conditions were examined in the study: small, medium, and large. The different levels of ICCs were simulated by varying the size of both Ψ_*B*_*j*_1___ and Ψ_*B*_*j*_2___ between-level crossed factor variances of FB1 and FB2, respectively. Both crossed factor variances were set to be the same with three different levels: 0.10, 0.25, and 0.50 while the within-level factor variance (Ψ_*w*_) was always fixed (1.00). Based on Equation (6b), these combinations of variances resulted in three different levels of ICCs: 0.08, 0.17, and 025 for small, medium, and large ICCs, respectively. For the residual variance, the within-level was set to 0.25 and the between-level was set to 0.05. Given the simulated factor variances, factor loadings, and residual variances, the item-level ICCs ranged 0.08–0.13 for small ICC, 0.13–0.25 for medium ICC, and 0.20–0.46 for large ICC. These ICC levels represent common situations encountered in educational research with multilevel data (Hox and Maas, 2001; Maas and Hox, 2005). Especially, when ICC is low, the statistical power for detecting the non-invariant factor loading became low even with a large number of clusters (Kim et al., 2012a).

By combining the two study conditions (magnitude of non-invariant factor loading and ICC), a total of 9 (3 × 3) scenarios were investigated in the study. We generated 1000 replications for each scenario.

#### Multiple-group MCFA

The generated data sets with cross-classified MCFA of two groups were then analyzed using the conventional multiple-group MCFA (i.e., the misspecified model) by ignoring one of the crossed factors (i.e., FB2) and treating the data as hierarchical multilevel data. To explore the performance of the conventional multiple-group MCFA with cross-classified data, we used the Type = TWOLEVEL routine in M*plus*, recommended by Kim et al. (2012a) for the conventional multilevel data. When target groups are at the between-level, the TYPE = TWOLEVEL routine decomposes the variance and covariance matrix into within- and between- models for the analysis. Thus, the within- and between-level variance components can be separately investigated. All data analyses were conducted using M*plus* 7.4. Supplementary Material provides the M*plus* scripts that were used for analyzing MCFA.

#### Model selection criteria

##### Chi-square difference test (Δ χ^2^)

For factorial invariance testing, we conducted the likelihood ratio test between a baseline model (i.e., configural invariance model) and sequentially more restricted invariance model (i.e., weak invariance model) under the null hypothesis of no difference between two models. When the null hypothesis is failed to reject (non-significant at α = 0.05), we conclude that the more restricted invariance model holds under study, indicating that weak invariance holds. Conversely, when the null hypothesis is rejected (significant at α = 0.05), we conclude that the less restricted invariance model holds under study, indicating the presence of a non-invariant factor loading.

The maximum likelihood estimation with robust standard errors (MLR) is employed as an estimator for continuous variables in the study. The MLR yields a robust chi-square test (Kaplan et al., 2009) by utilizing robust standard errors and a mean-adjusted chi-square statistic test. For comparing two competing models, the MLR requires the Satorra-Bentler scaled chi-square difference test (Satorra and Bentler, 1994; Brown, 2006; Heck and Thomas, 2009).

##### Goodness-of-fit indices

Considering the sensitivity of the χ^2^ test statistic to sample size, we have additionally examined the performance of the following difference (Δ) of the goodness-of-fit indices in comparing the two competing invariance models (configural versus metric): (a) IC (i.e., Δ AIC and Δ BIC; (b) Δ SRMR between and within; (c) Δ CFI; and (d) Δ RMSEA. The recommended cutoff values for determining the goodness-of-fit indices for the metric invariance model over the configural invariance model are: both ΔAIC and ΔBIC ≤ 4 (Burnham and Anderson, 2002), ΔSRMR ≤ 0.01 (Chen, 2007), ΔCFI ≤ 0.01 (Cheung and Rensvold, 2002), and ΔRMSEA ≤ 0.015 (Chen, 2007). Under the non-invariance conditions, if the Δ fit-index was smaller than the cutoff value, this indicated a miss/failure in detecting the non-invariance. Conversely, when the obtained value was larger than the cutoff value, this indicated a hit/success in detecting the non-invariance.

#### Analysis of simulation results

We examined the statistical power and Type I error rate of the chi-square difference test and goodness-of-fit indices and the relative bias of the parameter of interest to explore the performance of multiple-group conventional MCFA (ignoring a crossed factor of cross-classified multilevel data and treating the data as hierarchical data) in detecting the between-level non-invariant factor loading.

##### Type I error rate

For the invariant condition, Type I error rate was examined. Type I error referred to the proportion of the cases in which the Δ χ^2^ test falsely detected invariance as non-invariance over 1000 replications. The invariance at factor loading across groups should lead to failing to the rejection of equal factor loadings across groups (metric invariance) in the Δ χ^2^ tests of the misspecified model (multiple-group conventional MCFA).

##### Statistical power

Under the non-invariant conditions, we expected the null hypothesis of the Δ χ^2^ test to be rejected because one of the between-level factor loadings was simulated to be different (or non-invariant) across groups. The (empirical) statistical power rate was defined as the proportion of the cases in which the non-invariance at the between-level factor loading was correctly detected through the chi-square difference (Δ χ^2^) test and the Δ goodness of fit indices when using the conventional MCFA for testing factorial invariance.

##### Relative bias

The relative bias of the factor loadings of the non-invariant indicator (i.e., λ_*w*_ at the within-level; λ_*B*_*j*_1___ at the between-level) and the factor variance at each level (i.e., Ψ_*w*_ at the within-level; Ψ_*B*_*j*_1___ at the between-level) in multiple-group conventional MCFA (misspecified Model) were examined. For the non-invariance condition, we set one of the between-level factor loadings to be different across groups for one of the crossed factors. The target parameter of the relative bias is the between-level factor loading (λ_*B*_*j*_1___) of one group (group 2 in the current study) which was set to be smaller (i.e., non-invariant) than the factor loading of the other group (group 1 in the current study). The relative bias of DIF was also examined. The group difference (i.e., group 1–group 2) in the target between-level factor loading λ_*B*_*j*_1___ is the size of non-invariance (DIF). For estimating the relative bias, we used the group mean estimates across the replications from configural invariance model. The relative bias of the estimates was computed using the following equation:

$$\begin{array}{l} B\left(\hat{\beta }\right)=\frac{\hat{\beta }-\beta }{\beta } \end{array}$$

where $\hat{\beta }$ was the parameter estimate across the valid replications in the misspecified model, and β was the population parameter. To evaluate the estimated relative bias, we applied cutoffs of 0.05 for the loading estimates and of 0.10 for the factor variance estimates, which have been recommended as the acceptable magnitude of relative bias (Hoogland and Boomsma, 1998). That is, when relative bias is below 0.05 and 0.10 for the loading estimates and for the factor variance estimates, respectively, the parameter estimate of interest is considered unbiased. A positive relative bias indicates an overestimation of the target parameter (i.e., factor loading and factor variance in this simulation study), whereas a negative relative bias indicates an underestimation of the target parameter.

### Results

Table 2 presents the empirical statistical power rate for the non-invariant models in conventional MCFA. Table 3 depicts the relative bias for the target factor loading estimates and factor variance estimates in conventional MCFA. All MCFA models were successfully analyzed.

**Table 2:** **Summary of empirical power rate of Satorra-Bentler chi-square difference test (Δ χ^2^) and Δ goodness-of-fit indices in factorial invariance testing using multiple-group MCFA (misspecified model)**.

**Simulation design factors**									
**Intra-class correlation (ICC)**	**Small**			**Medium**			**Large**		
**Difference in Factor Loading at between-level (DIF)**	**Small (0.15)**	**Medium (0.25)**	**Large (0.35)**	**Small (0.15)**	**Medium (0.25)**	**Large (0.35)**	**Small (0.15)**	**Medium (0.25)**	**Large (0.35)**
**Δ χ^2^ TEST** ***(df** = **3*****)**									
Mean Δ χ^2^	0.06	0.12	0.19	0.18	0.44	0.80	0.57	1.52	2.73
Standard deviation	0.07	0.12	0.21	0.16	0.41	0.74	0.49	1.38	2.35
Empirical power rates	0.00	0.00	0.00	0.00	0.00	0.00	0.00	0.00	0.04
**Δ GOODNESS-OF-FIT INDICES EMPIRICAL POWER RATES**									
Δ AIC	0.00	0.00	0.00	0.00	0.00	0.00	0.00	0.00	0.00
Δ BIC	0.00	0.00	0.00	0.00	0.00	0.00	0.00	0.00	0.00
Δ CFI	0.00	0.00	0.00	0.00	0.00	0.00	0.00	0.00	0.00
Δ RMSEA	0.00	0.00	0.00	0.00	0.00	0.00	0.00	0.00	0.00
Δ SRMR Within	0.00	0.00	0.00	0.00	0.00	0.00	0.00	0.00	0.00
Δ SRMR Between	0.00	0.00	0.00	0.00	0.00	0.01	0.00	0.00	0.01

*For factorial invariance testing, we used the conventional multiple-group multilevel CFA with Type = TWOLEVEL routine in Mplus 7.4 and compared between configural invariance model and metric invariance model. For simulation design factors, the three different levels of ICC, 0.08, 0.17, and 0.25 are corresponding to small, medium and large levels; the three different magnitudes of non-invariant factor loading, 0.15, 0.25, and 0.35 are corresponding to small, medium and large loading difference between two groups. In Δ χ^2^ test, Mean is the average of Satorra-Bentler Chi-square difference test statistics across 1000 replications; Standard Deviation in the corresponding standard deviation of Δ χ^2^ test; Empirical power rate is the rejection rate. As for goodness-of-fit Indices, Akaike- and Bayesian-information criteria (AIC and BIC, respectively); comparative fit index (CFI); the root mean square error of approximation (RMSEA); and the standardized root mean square residual (SRMR) within and between were used*.

**Table 3:** **Relative bias in factor loading and factor variance in the configural invariance model (misspecified conventional MCFA)**.

**Simulation design factors**		**Factor loading**								**Factor variance**					
		**Within**			**Between**			**Difference in** λ_*****Bj***1**_ **(**Δ **G1-G2)**		**Within**			**Between**		
**ICC**	**DIF**	**RB PE**	**RB SE**	**RMSE**	**RB PE**	**RB SE**	**RMSE**	**RB PE**	**RMSE**	**RB PE**	**RB SE**	**RMSE**	**RB PE**	**RB SE**	**RMSE**
Small	Small	0.00	0.03	0.02	0.20	0.06	0.17	−0.95	0.02	0.09	0.03	0.10	−0.08	0.22	0.09
	Medium	0.00	0.03	0.02	0.37	0.06	0.26	−0.95	0.02	0.09	0.03	0.10	−0.14	0.23	0.09
	Large	0.00	0.03	0.03	0.62	0.06	0.29	−0.96	0.03	0.09	0.03	0.09	−0.20	0.25	0.08
Medium	Small	0.00	0.04	0.02	0.17	0.07	0.15	−0.85	0.03	0.23	0.03	0.24	−0.05	0.17	0.09
	Medium	0.00	0.04	0.02	0.33	0.07	0.23	−0.85	0.04	0.23	0.03	0.24	−0.10	0.19	0.09
	Large	0.00	0.04	0.02	0.54	0.08	0.31	−0.85	0.06	0.23	0.03	0.24	−0.13	0.20	0.10
Large	Small	0.00	0.04	0.02	0.15	0.09	0.13	−0.71	0.05	0.46	0.06	0.47	−0.04	0.18	0.17
	Medium	0.00	0.04	0.02	0.28	0.09	0.19	−0.71	0.08	0.46	0.06	0.47	−0.06	0.18	0.18
	Large	0.00	0.04	0.02	0.46	0.09	0.26	−0.71	0.11	0.46	0.06	0.47	−0.07	0.18	0.18

*For Simulation design factors, ICC is intra-class correlation; DIF is the difference in a target factor loading (λ_Bj1_) at the between-level across two groups. G1 is group 1 as a reference group; G2 is group 2 as a focal group where the factor loading of one item was set to be smaller than the factor loading of G1 in the study. λw and λ_Bj1_ are the estimated parameter of the target factor loading at the within- and between-level (remaining), respectively. Ψw and Ψ_Bj1_ are the factor variance at the within- and between-level (remaining), respectively. RB PE is the relative bias of parameter estimates. RB SE is the relative bias of the standard errors of the corresponding parameter. RMSE is the root mean square error of parameter estimates*.

### Empirical statistical power for detecting non-invariance

For the invariant model, the Type I error rates with the Satorra-Bentler chi-square difference test (Δ χ^2^) and the Δ goodness of fit indices were all zero for all study conditions and were not tabled to save space. For the non-invariant model, Satorra-Bentler chi-square difference test (Δ χ^2^) and Δ goodness-of-fit indices showed extremely low power rates (zero or close to zero). The average of Δ χ^2^ statistics and the corresponding standard deviation of the Δ χ^2^ statistics across valid replications are also reported in Table 2. In summary, when the between-level factor loadings were invariant across groups, conventional MCFA performed very well in terms of Type I error even though one of the crossed factors at the between level was completely ignored and the data were treated as strictly hierarchical. On the contrary, when the crossed factors were not fully considered and the cross-classified data were treated as strictly hierarchical with the presence of non-invariance at between-level factor loadings across groups, MCFA performed very poorly with zero or near zero power rates regardless of the size of non-invariance and the level of ICC.

### Relative bias of parameter estimates

Table 3 summarizes the relative bias of parameter estimates (RB PE), the relative bias of the standard error of corresponding parameter estimates (RB SE), and root mean squared error (RMSE) for the target factor loading estimates (i.e., λ_*w*_ at the within-level; λ_*B*_*j*_1___ at the between-level) and the factor variance estimates (i.e., Ψ_*w*_ at the within-level; Ψ_*B*_*j*_1___ at the between-level) in group 2 (focal group; set to be smaller than reference group 1). For the standard deviation of bias of the parameter estimates, we reported RMSE. Furthermore, for an evaluation of the quality of the standard errors, the relative bias of the standard error of the parameter estimates (RB SE) was computed by quantifying how large the difference is between the mean estimated standard error and the standard deviation of the parameter estimates across replications (Hoogland and Boomsma, 1998; Cheung and Chan, 2005). The relative bias below 0.10 in their standard errors indicates good estimation methods used for the study (Hoogland and Boomsma, 1998). The relative bias of the parameter estimates of interest for group 1 was not reported for the simplicity of the table because the relative bias were almost zero for all study conditions. In addition, the relative bias of the non-invariance in λ_*B*_*j*_1___ across groups (group 1–group 2) and corresponding RMSE were reported. The relative bias was estimated only for the non-invariant conditions.

#### Difference in target non-invariant factor loading estimates between groups (DIF)

The parameter of the between-level factor loading λ_*B*_*j*_1___ in group 2 appears overestimated in MCFA. The relative bias of λ_*B*_*j*_1___ ranged from 0.46 to 0.67 for large DIF, from 0.28 to 0.37 for the medium DIF, and from 0.15 to 0.20 for small DIF. Because no relative bias of λ_*B*_*j*_1___ was observed in group 1, the overestimated parameter estimates of λ_*B*_*j*_1___ in group 2 led to the underestimated DIF in λ_*B*_*j*_1___. This underestimation of DIF became more serious as the ICC decreased. The relative bias ranged from −0.71 to −0.96 and was above the cutoff in the absolute value (i.e., 0.05). In contrast, the parameter estimates of the within-level factor loading λ_*w*_ in both group 1 and group 2 were unbiased regardless of study conditions. No substantial relative bias in the standard errors of λ_*w*_ and λ_*B*_*j*_1___ was observed for all study conditions.

#### Factor variance

The relative biases of the factor variance at the within level (Ψ_*w*_) were almost identical across two groups for all study conditions. The within factor variance Ψ_*w*_ was generally overestimated and the greater overestimation was associated with the larger ICC. On the other hand, the between factor variance Ψ_*B*_*j*_1___ in group1 was positively biased whereas the between factor variance Ψ_*B*_*j*_1___ in group 2 was negatively biased (i.e., underestimated by relative bias 0.10–0.20 above the cutoff of 0.10) when the DIF size was medium and large. This underestimation of Ψ_*B*_*j*_1___ in group 2 became serious as the ICC decreased. In addition, the overestimation of the standard error of Ψ_*B*_*j*_1___ became serious as the ICC decreased.

## Study 2: testing factorial invariance using cross-classified multiple indicators multiple causes (MIMIC) model with cross-classified data

We examined cross-classified multiple indicators multiple causes (MIMIC) model to fit a correct model in testing measurement invariance with cross-classified data using M*plus* (version 7.4). MIMIC modeling (Jöreskog and Goldberger, 1975; Muthén, 1989) is one of several methods used to test factorial invariance and population heterogeneity (e.g., Kim et al., 2012b). MIMIC models employ an observed variable as a covariate of latent factors. The inclusion of a grouping covariate allows testing a group difference via a regression-type analysis.

Testing factor loading invariance that Study 1 focused on requires a grouping covariate and its interaction with the between-level factor. However, this type of interaction terms is not allowed with the Bayesian estimator which is the default estimator with cross-classified data in the current software (i.e., M*plus version 7.4)*. Therefore, in Study 2, we tested cross-classified MIMIC model to examine the feasibility of testing intercept invariance with cross-classified data. Supplementary Material provides the M*plus* script that was used for analyzing cross-classified MIMIC.

### Methods

#### Data generation and simulation design factors

We simulated the intercept at the between-level of one target item to be different across two groups with 500 replications. The size of non-invariance associated with the intercept was 0.25 and 0.50, representing small and large non-invariance (i.e., DIF). Other than the parameter of non-invariance, two groups have the identical population parameters. Two levels of ICC, namely 0.08 and 0.25 for small and large levels, respectively were simulated. Thus, when intercept non-invariance was simulated to evaluate power in detecting non-invariance, 4 conditions (2 ICC × 2 DIF size) were created. In addition, complete invariance conditions with two ICC levels (i.e., small and large ICC with invariant intercepts) were generated to examine Type I error.

#### Analytic procedures

Instead of modeling a separate MCFA for each group, cross-classified MIMIC constructs a single model with a grouping variable as a covariate, assuming strict invariance between two groups. To test the intercept invariance, the assumption of strict invariance should be relaxed by allowing a difference in the intercept between groups. Thus, a set of nested cross-classified MIMC models are typically analyzed. First, one direct effect (i.e., the target item regressed on the grouping covariate) of one crossed factor (School in the study) is constructed to test the factorial invariance of the intercept. The model with one direct effect on the target item (i.e., relaxed model) and the model in which the direct effect was constrained to be zero assuming invariance of the intercept (i.e., constrained model) are compared. The statistical support of the relaxed model with the direct effect indicates the non-invariance of the target intercept. Of note is that to evaluate the feasibility of the cross-classified MIMIC model for factorial invariance testing with cross-classified data, we investigated the significance and magnitude of the direct effect in the relaxed model instead of conducing the model comparison between the constrained and relaxed mdoels. We consider the statistical significance of the direct effect in the relaxed model at α = 0.05 as the presence of non-invariance in the intercept (i.e., the intercepts are statistically significantly different between two groups). Thus, with the intercept non-invariance conditions, the proportion of cases in which the direct effect from the grouping covariate to the target item was statistically significant at α = 0.05 was considered as power; the same proportion was considered as Type I error with the invariance conditions. In addition, the relative bias for the magnitude of the direct effect as well as the factor variance at each level was evaluated.

### Results

First, the Type I error rates for invariant models and power rates for non-invariant models in detecting the group difference (i.e., direct effect of a grouping covariate on the target non-invariant item) using cross-classified MIMIC are presented in Table 4 along with the parameter estimates of the direct effect. When there was invariance for the between-level intercept, Type I error was almost zero across valid replications regardless of ICC. When there was non-invariance for the between-level intercept only, the power was 1 in the large ICC conditions and close to 1 (0.975 and 0.994 for the small and large DIF, respectively) in the small ICC conditions. In summary, the cross-classified MIMIC with a grouping covariate appeared adequate for detecting non-invariance in the between-level intercept only regardless of the size of DIF and ICC and yielded unbiased estimates of the direct effect.

**Table 4:** **Type I error and power of cross-classified multiple indicators multiple causes (MIMIC) models to detect intercept non-invariance**.

**Simulation design factors**		**Direct effect (group difference on a target item)**			**Proportion in detecting group difference**	**Valid replications**
**DIF**	**ICC**	**Population parameter (DIF)**	**Parameter estimates**	***p*-value (SE)**		
None	Small	0.00	0.00	0.479	0.000	487
				(0.018)		
	Large	0.00	0.00	0.479	0.000	500
				(0.015)		
Small	Small	0.25	0.25	0.008	0.975	487
				(0.017)		
	Large	0.25	0.25	0.005	1.000	500
				(0.005)		
Large	Small	0.50	0.50	0.001	0.994	500
				(0.008)		
	Large	0.50	0.50	0.000	1.000	500
				(0.000)		

*To fit the correct model, we used cross-classified MIMIC modeling with Type = CROSSCLASSIFIED routine in Mplus 7.4 using cross-classified data. ICC is intra-class correlation. DIF is the difference in a target intercept at the between-level across two groups. For simulation design factors, the two different levels of ICC, 0.08 and 0.25 are corresponding to small and large levels; the two different magnitudes of non-invariant intercept, 0.25, and 0.50 are corresponding to small and large intercept difference between two groups. SE is the average standard error of the corresponding estimates. We consider the statistical significance of the direct effect in the relaxed model at α = 0.05 as the presence of non-invariance in the intercept (i.e., the intercepts are statistically significantly different between two groups). Thus, with the intercept non-invariance conditions (i.e., DIF = Small and Large), the Proportion in Detecting Group Difference was considered as power; the same proportion was considered as Type I error with the invariance conditions (i.e., DIF = None)*.

Second, the parameter estimates and relative bias of factor variance at the within- and the between-level are presented in Table 5. The within-level factor variance parameters were unbiased across all study conditions whereas the between-level factor variances for two crossed factors were slightly biased. The school-level factor variance where the group difference was constructed was slightly underestimated (e.g., relative bias of −0.12 above the cutoff in absolute value of 0.10 in small ICC conditions) whereas the neighborhood-level factor variance where no group difference was present was slightly overestimated (e.g., relative bias of 0.10 above the cutoff in absolute value of 0.10 in large ICC conditions), but overall the factor variance estimates appear unbiased given the raw bias was not substantial (e.g., 0.01, 0.03, and 0.05). Because the performance of cross-classified MIMIC is unknown, issues related to the direction of the bias call for further investigation.

**Table 5:** **Parameter estimates and relative bias of factor variance using cross-classified multiple indicators multiple causes (MIMIC) models with a grouping variable as a covariate**.

**Simulation conditions**		**Population parameters**			**Parameter estimates**			**Relative bias of parameter estimates**		
**DIF**	**ICC**	**Within level**	**Neighborhood level**	**School level**	**Within level**	**Neighborhood level**	**School level**	**Within level**	**Neighborhood level**	**School level**
None	Small	1.00	0.10	0.10	0.99	0.11	0.09	−0.01	0.07	−0.09
	Large	1.00	0.50	0.50	0.99	0.55	0.47	−0.01	0.10	−0.05
Large	Small	1.00	0.10	0.10	0.99	0.11	0.09	−0.01	0.07	−0.12
	Large	1.00	0.50	0.50	0.99	0.55	0.47	−0.01	0.10	−0.05
Small	Small	1.00	0.10	0.10	0.99	0.11	0.09	−0.01	0.07	−0.12
	Large	1.00	0.50	0.50	0.99	0.55	0.47	−0.01	0.10	−0.05

*ICC is intra-class correlation. DIF is the difference in a target intercept at the between-level across two groups. Population parameters are the generated factor variances in the simulation. Parameter estimates are the average of the estimated factor variances across valid replications*.

## Discussion

Testing measurement invariance is a very important step before one can meaningfully compare the (mean) difference on a latent construct or the corresponding composite score between groups. Measurement invariance testing can be utilized to examine possible differences between groups at the organizational units of a particular measure. For between-level grouping comparison, more complexity arises with factorial invariance (FI) testing in cross-classified multilevel data due to the multiple crossed factors compared to FI testing in conventional multilevel data. Ideally, the multiple crossed factors should be taken into account when conducting FI test. However, to date, no statistical program can fully conduct FI testing and take into account the cross-classified data structure simultaneously. For this reason, researchers possibly treat cross-classified data as a conventional multilevel data (i.e., as strictly nested or hierarchical) by ignoring one of the crossed factors. Hence, it is important to examine the potential impact of ignoring the cross-classified data structure in FI testing.

According to the simulation results, we found two biases in multiple group MCFA when misanalysing the partially cross-classified multilevel data. Due to the redistribution of variance component mechanism (Luo and Kwok, 2009), the variance of the ignored crossed factor (Ψ_*B*_*j*_2___, between-level) redistributes to the lower level and results in substantial overestimation of the variance component at the lower level (Ψ_*w*_, within-level) whereas the remaining between level factor variance Ψ_*B*_*j*_1___ in the focal group is slightly underestimated. The combination of the substantially o*verestimated* (*inflated*) Ψ_*w*_ and the slightly *underestimated* Ψ_*B*_*j*_1___ results in an underestimated ICC in multiple-group MCFA given that ICC is computed by using the total variability, the sum of factor variance components (Ψ_*B*_*j*_1___ at the between-level and Ψ_*W*_ at the within-level) as a denominator and Ψ_*B*_*j*_1___, as a numerator. As shown by Kim et al. (2012a), ICC is related to the statistical power for detecting non-invariant factor loadings when testing factorial invariance in multilevel data and lower ICC is typically linked to lower statistical power even under large sample size conditions and a correctly specified MCFA model. Given the relation between ICC and the power, the underestimation of ICC due to the variance redistribution in the misspecified multiple-group MCFA possibly leads to low power of detecting factor loading non-invariance at the between level.

In addition to ICC, we found the bias in the magnitude of non-invariance/difference in the target factor loadings between the two groups at the between-level λ_*B*_*j*_1___. Under the non-invariant conditions, the target between-level factor loading in one of the groups (i.e., λ_*B*_*j*_1___ for reference group, group 1) was set to 0.90 while the same factor loading for the other group (i.e., λ_*B*_*j*_1___ for focal group, group 2) was set to be smaller: 0.55, 0.65, or 0.75 to represent for large (0.35), medium (0.25), and small (0.15) differences, respectively. Nevertheless, given that no relative bias of λ_*B*_*j*_1___ for group1 was found, the positively biased λ_*B*_*j*_1___ for group 2 led to reducing the differences between the two groups, compared with the originally simulated differences. Hence, given all other conditions were held constant, the reduction in the difference between the non-invariant loadings would result in lower statistical power to detect such diminished effect and failure in detecting the violation of metric invariance when the crossed factors were not fully taken into account in FI testing. Note that we tested the factor loadings of all items simultaneously for metric invariance. If the factor loading of each item is tested one by one, it might be possible to improve the power, but this item-level analysis was not investigated in this study.

Furthermore, the investigation on the performance of cross-classified MIMIC in detecting the group difference at the between-level intercept only revealed that cross-classified MIMIC with a grouping covariate appears adequate for invariance testing. We examined the cross-classified MIMIC models that included one direct effect (i.e., the target item regressed on a grouping covariate) at only one of crossed factors (e.g., school-level). In the cross-classified MIMIC, the overestimation of within-level factor variance of one group due to variance redistribution did not occur. The power rates to detect the non-invariance in the between-level intercept only were also very high.

In summary, according to the findings from our simulation study, when the between-level factor loadings are invariant across the between-level comparison groups, conventional MCFA appears to perform well for cross-classified multilevel data even though a crossed factor was omitted in testing measurement invariance. On the other hand, for the non-invariant conditions two potential sources of bias (i.e., the *underestimated* ICC and the *underestimated* factor loading difference) might lead to the low statistical power in detecting the between-level factor loading non-invariance when researchers misanalyze the cross-classified multilevel data using multiple-group MCFA. The failure to detect the non-invariant factor loading resulted in concluding the non-invariant model as invariant between groups. In conclusion, given the redistribution of variance components and underestimation of the size of factor loading difference, the conventional multilevel CFA is not recommended for factorial invariance testing for cross-classified multilevel data.

### Limitation and directions for future research

Our current findings need to be interpreted given certain limitations. First, sample size was not considered as a simulation factor in our simulation study. Second, to mimic more realistic education settings, the current study followed Luo and Kwok's (2009) simulation study and created the partial cross-classified data structure. Fifty neighborhoods (feeder, FB2) nested within 20 schools (receiver, FB1) were created and students were cross-classified by schools and neighborhoods. For future research different levels of partial cross-classification can be considered by combining two conditions: (a) the number of feeder (neighborhoods) selected as cross-classified and (b) the number of receivers (schools) assigned as cross-classified. In other words, we can create a more cross-classified data structure by increasing the selected number of feeder (neighborhoods, FB2) or receiver (schools, FB1).

Third, in this study, the research scenario for the misspecified model (MCFA) focused on the invariance at the factor loading only. In practice, non-invariance may exist for other parameters such as intercepts or both factor loadings and intercepts. In addition, non-invariance can occur at both within and between models simultaneously. Moreover, the structures of the within- and between-models may not always be identical. Overall, the performances of MCFA need to be studied under these more complex research settings with various sources of non-invariance.

Furthermore, due to the limitation of software, the research scenario for a correct model (cross-classified MIMIC) only focused on the invariance at the intercept only. Future research on the performances of cross-classified MIMC would be very beneficial in testing factorial invariance with cross-classified data. In addition, further development of software for such model (i.e., multiple group cross-classified SEM) or alternative option (e.g., cross-classified MIMIC with a factor by covariate interaction at the between level) for analyzing this type of data is needed.

## Author contributions

All authors listed, have made substantial, direct and intellectual contribution to the work, and approved it for publication.

### Conflict of interest statement

The authors declare that the research was conducted in the absence of any commercial or financial relationships that could be construed as a potential conflict of interest.
