# Supplementary material for: Impact of Not Addressing Partially Cross-Classified Multilevel Structure in Testing Measurement Invariance: A Monte Carlo Study
Source: Front Psychol. 2016 Mar 23;7:328. doi: 10.3389/fpsyg.2016.00328 (PMC4804162; doi:10.3389/fpsyg.2016.00328)
Supplement: Supplementary file 1 [file DataSheet1.docx]

**Appendix A**

*Summary of Similarities and Differences between Cross-Classified MCFA and Conventional MCFA*

| Model | Cross-Classified MCFA | Conventional MCFA |
| --- | --- | --- |
| Latent factor means: $\eta$ | ${\eta_{i(j_{1}j_{2})}=\alpha}_{{Bj}_{1}}+\alpha_{{Bj}_{2}} + \eta_{wij}+ \eta_{{Bj}_{1}}+ \eta_{{Bj}_{2}}$ | $\eta_{ij}=\alpha+ \eta_{wij}+ \eta_{Bj}$ |
| Variance of the factor$: V \left( \eta\right)$ | $\Psi_{T}= \Psi_{W}+ \Psi_{{Bj}_{1}}+ \Psi_{{Bj}_{2}}$ | $\Psi_{T}= \Psi_{W}+ \Psi_{B}$ |
| Variance of observed scores:$V \left( y \right)$ | $\Sigma_{T}= {\Sigma_{w}+ \Sigma}_{{Bj}_{1}}+\Sigma_{{Bj}_{2}}$ | $\Sigma_{T}= \Sigma_{w}+ \Sigma_{B}$ |
| Variance of unique factor: $V \left( \varepsilon\right)$ | $V \left( \varepsilon_{i(j_{1}j_{2})} \right)= \Theta_{W} + \Theta_{{Bj}_{1}} +\Theta_{{Bj}_{2}}$ | $V \left( \varepsilon_{ij} \right)= \Theta_{W}+ \Theta_{B}$ |
| Measurement model: *X* | $X_{i(j_{1}j_{2})}=\tau_{{Bj}_{1}}+\tau_{{Bj}_{2}}$  $+ {\Lambda_{{Bj}_{1}}\eta}_{{Bj}_{1}}+{\Lambda_{{Bj}_{2}}\eta}_{{Bj}_{2}}{+ \Lambda_{W}\eta}_{Wij}$  $+ \varepsilon_{{Bj}_{1}}+\varepsilon_{{Bj}_{2}}+\varepsilon_{wij}$ | $X_{ij}=\tau_{B}$  ${{{+ \Lambda_{W}\eta}_{Wij} + \Lambda}_{B}\eta}_{Bj}$  $+ \varepsilon_{wij}+\varepsilon_{Bj}$ |
| Covariance structure of measurement model:$\Sigma$ | $\Sigma_{{Bj}_{1}}=$ ${\Lambda_{{Bj}_{1}}\Psi}_{{Bj}_{1}}\Lambda_{{Bj}_{1}}^{'}+ \Theta_{{Bj}_{1},}$  $\Sigma_{{Bj}_{2}}=$ ${\Lambda_{{Bj}_{2}}\Psi}_{{Bj}_{2}}\Lambda_{{Bj}_{2}}^{'}+ \Theta_{{Bj}_{2},}$  $\Sigma_{W} = {\Lambda_{W} \Psi}_{W}\Lambda_{W}^{'} + \Theta_{W}$ | $\Sigma_{B}= {\Lambda_{B}\Psi}_{B}\Lambda_{B}^{'} + \Theta_{B}$  $\Sigma_{W}={\Lambda_{W}\Psi}_{W}\Lambda_{W}^{'}+ \Theta_{W}$ |

*Note:* *i* indexes a within-level unit, $j_{1}$ and $j_{2}$ indexes a cluster of the crossed factors (e.g., schools and neighborhoods). $\alpha$ is the grand mean of $X_{ij}$. $\tau$ is a vector of intercepts. $\Lambda$ is a matrix of factor loadings. $\eta$is a matrix of latent or common factor scores. $\varepsilon$ is a matrix of unique factor scores or residuals. 𝛹 is the factor variance. $\Theta$ is the residual variance.

**Appendix B**

**Mplus script for data generation**

Montecarlo:

names are y1-y4;

nobservations = 10000;

csizes = 50 [20 (10)];

seed = 12313000;

ncsize = 1[1];

nreps = 1000;

Analysis:

type = crossclassified;

estimator = bayes;

proc = 2;

Model population:

%within%

fw by y1@1 y2@.9 y3@.7 y4@.8;

fw@1;

[fw@0.5];

y1-y4*.25;

%between level2a%

fa by y1@1 y2@0.75 y3@.7 y4@.8;

fa@.50;

[fa@0.0];

y1-y4*.05;

%between level2b%

fb by y1@1 y2@0.75 y3@.7 y4@.8;

fb@.50;

[fb@0.5];

y1-y4*.05;

Model:

%within%

fw by y1-y4;

fw@1;

[fw@0.5];

y1-y4*.25;

%between level2a%

fa by y1-y4;

fa@.50;

[fa@0.0];

y1-y4*.05;

%between level2b%

fb by y1-y4;

fb@.50;

[fb@0.5];

y1-y4*.05;

**Multilevel CFA model**

TITLE: Multilevel CFA for Configural invariance

VARIABLE:

names = y1-y4 school neighbor group;

usevariables = y1-y4 school group;

grouping is group (0=G1 1=G2);

cluster is school;

ANALYSIS: TYPE=TWOLEVEL;

MODEL:

%within%

fw by y1-y4;

%between%

fb by y1-y4

MODEL G2:

%between%

fb by y1@1 y2* y3* y4*; ! factor loadings of group 2 are relaxed

TITLE: Multilevel CFA for Weak invariance;

VARIABLE:

names = y1-y4 school neighbor group;

usevariables = y1-y4 school group;

grouping is group (0=G1 1=G2);

cluster is school;

ANALYSIS: TYPE=TWOLEVEL;

MODEL:

%within%

%within%

fw by y1-y4;

%between%

fb by y1-y4

**Cross-classified MIMIC model**

TITLE: Cross-classified MIMC for Relaxed Model;

VARIABLE:

Names = y1-y4 school neighbor group;

Usevariables = y1-y4 school group;

Cluster=school neighbor;

Between = (school) group;!grouping variable “group” is specified as a between-level covariate

ANALYSIS:

Type=CROSSCLASSIFIED RANDOM;

Estimator= Bayes;

Processors=2;

MODEL:

%WITHIN%

FW by y1@1 y2* Y3* y4*;

%Between school%

FB1 by y1@1 y2* Y3* y4*;

FB1 on g;

y2 on g; !test invariance of intercept of y2

%Between neighbor%

FB2 by y1@1 y2* Y3* y4*;

TITLE: Cross-classified MIMC for Constrained Model;

MODEL:

%WITHIN%

FW by y1@1 y2* Y3* y4*;

%Between school%

FB1 by y1@1 y2* Y3* y4*;

FB1 on g;

y2 on g@0; ! fix intercept invariance of y2 at zero

%Between neighbor%

FB2 by y1@1 y2* Y3* y4*;
